# Supplementary material for: Efficient pedigree recording for fast population genetics simulation
Source: PLoS Comput Biol. 2018 Nov 1;14(11):e1006581. doi: 10.1371/journal.pcbi.1006581 (PMC6233923; doi:10.1371/journal.pcbi.1006581)
Supplement: S1 Text — The supplementary text contains (A) benchmarking of run time and memory usage on simulations without selection; (B) benchmarking of memory usage with selection; (C) an analysis of the effect of simplification interval on run times; (D) details for the simuPOP implementation; and (E) more details, and a listing, of the simplification algorithm. (PDF) [file pcbi.1006581.s001.pdf]

A Timing and memory use with no selection

We also performed a more limited set of simulations without natural selection. For these we used `C++` as in the main text, but also provide results from our proof-of-concept implementation that uses `simuPOP` (see Appendix D).

The total run times are shown in Figure D1 and for `C++` show the same qualitative behavior as simulations with selection (Figure 1). The relative improvement due to pedigree tracking is again substantial in these simulations (Figure D2). In fact, we see more of a benefit to pedigree tracking here with `fwdpy11` than we did with selection (Figure 2). The reason is that the fitness function exits close to instantly in simulations without selection that are based on `fwdpp`, meaning that `fwdpy11` is doing little more than generating random numbers and book-keeping in Figures D1 and D2. For `simuPOP`, the results show a qualitatively similar pattern but (1) the simulations for $N = 10000$ or greater did no finish in the 72 hour limit or exceed memory limits (more than 500 GB), and (2) the magnitude of the speedup is much less. The total RAM use for simulations without selection is shown in Figure D3.

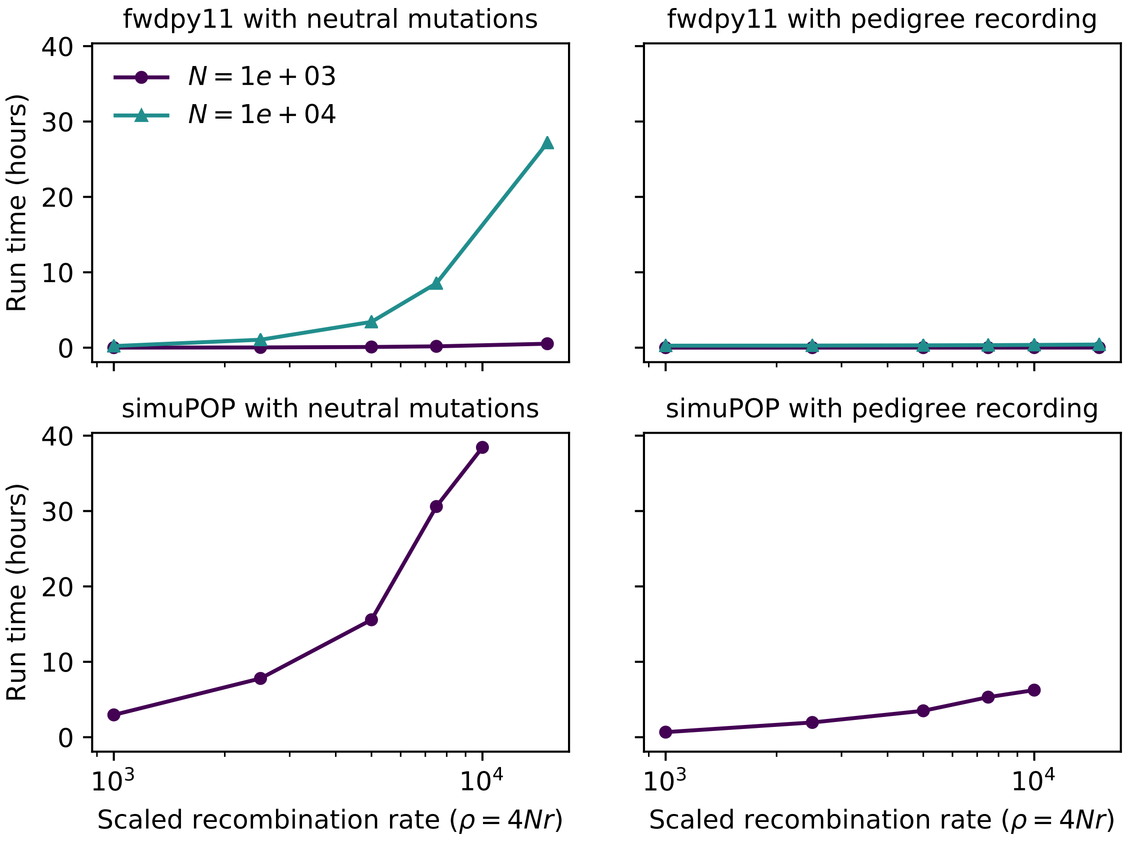

Figure D1: Total run time for a single simulation replicate as a function of region length, measured as the scaled recombination parameter $\rho = 4Nr$. The simulations here involve no natural selection.

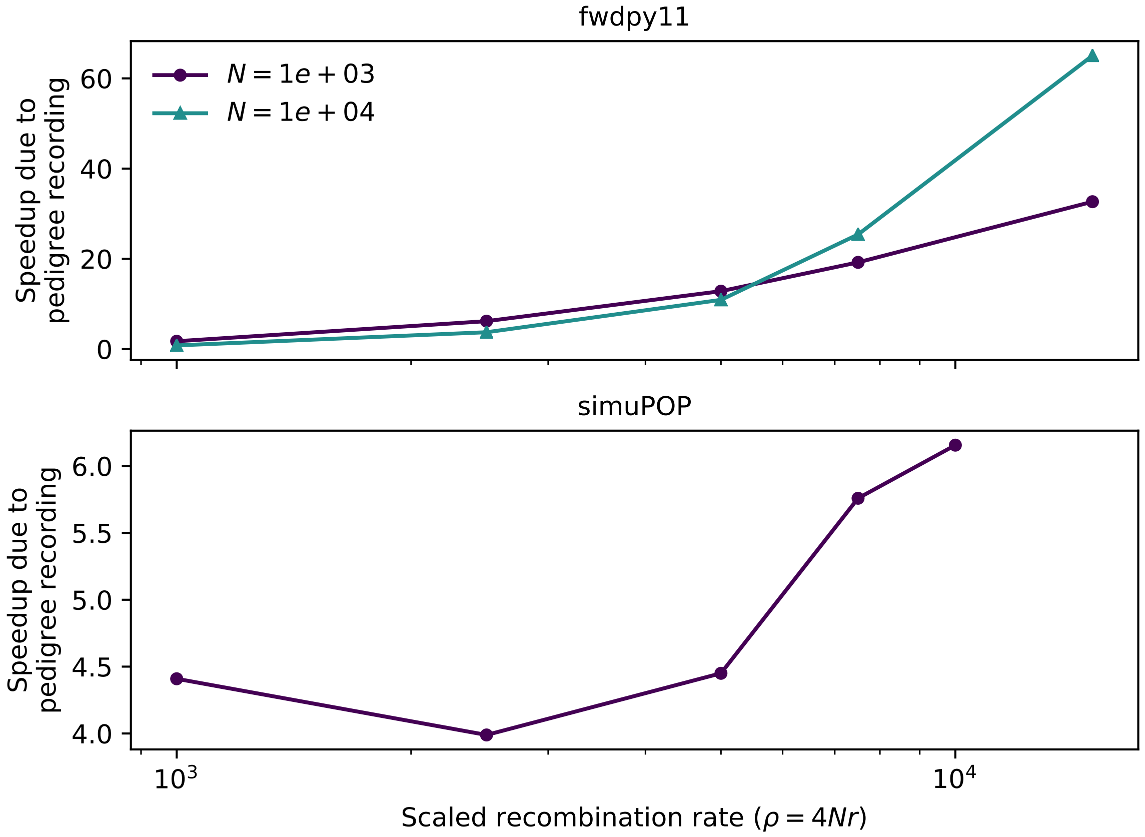

Figure D2: Relative performance improvement due to pedigree tracking for simulations without selection. Data points are taken from Figure D1 and show the ratio of run times tracking neutral variation over the run times tracking the pedigree.

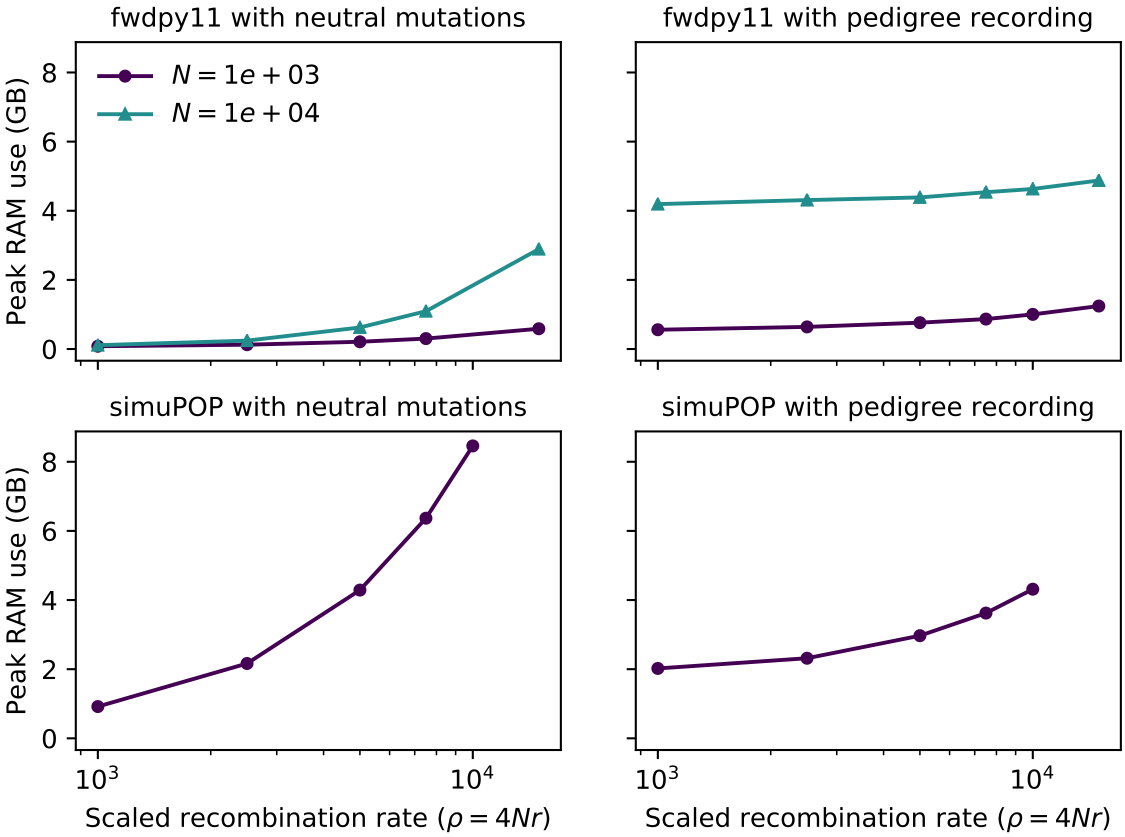

Figure D3: Peak RAM use in gigabytes (GB) for simulations without selection. The plots are from the same data as in Figure D1.

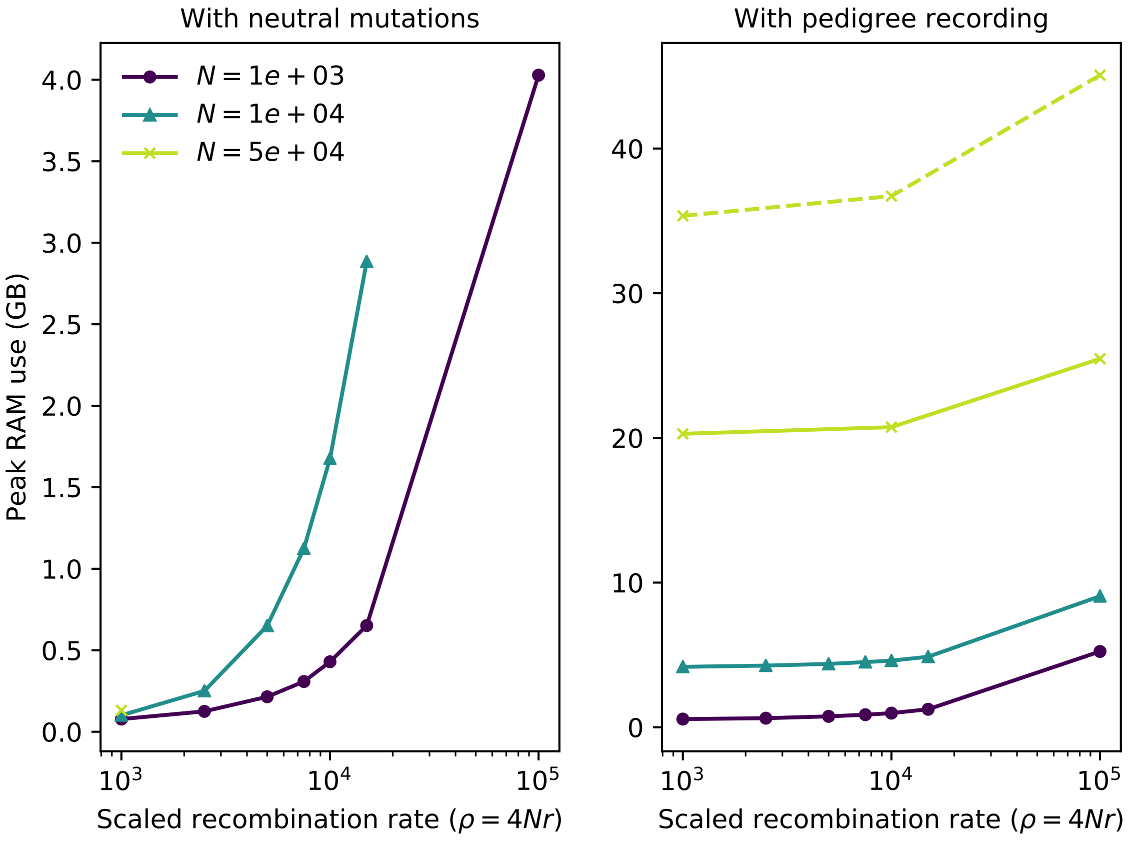

Figure B1: Peak RAM use in gigabytes (GB) for simulations with selection. The plots are from the same data as in Figure 1. Note that the y-axis scales differ considerably across panels.

B Memory usage with selection

The peak RAM use in gigabytes (GB) is shown for the simulations with selection in Figure B1. For our parameters, which involved simplifying every 10^3 generations, simulation while recording pedigrees uses more RAM than a standard simulation. This is because with pedigree recording, a non-recombinant individual still requires one new edge and two new nodes to be allocated. Without pedigree recording, no new memory is allocated for such an offspring by `fwdpp`. Thus, pedigree recording accumulates a large amount of data in RAM until simplification. This extra RAM consumption is the trade off for reduced run times and the degree of extra RAM needed can be reduced by simplifying more often. The simulations where we run the simplification step in a separate thread require even more RAM (dashed line in Figure B1) because up to four sets of unsimplified data are stored in RAM. In practice, the RAM consumption can be minimized by simplifying more often.

C Effect of simplification interval on run time and memory use

We performed a limited set of simulations to explore the effect of the simplification interval on run times and memory use. Figure C2 shows that run times increase for simplification intervals less than 100 generations.

For intervals longer than 100 generations, run times are very similar, with no appreciable difference between 10^3 and 10^4 generations. As expected, the simplification interval has a near-linear effect on peak RAM use (Figure C3). Taken together Figures C2 and C3 suggest that one can tune the simplification interval to available RAM with little effect on run times provided that simplification doesn't occur too often.

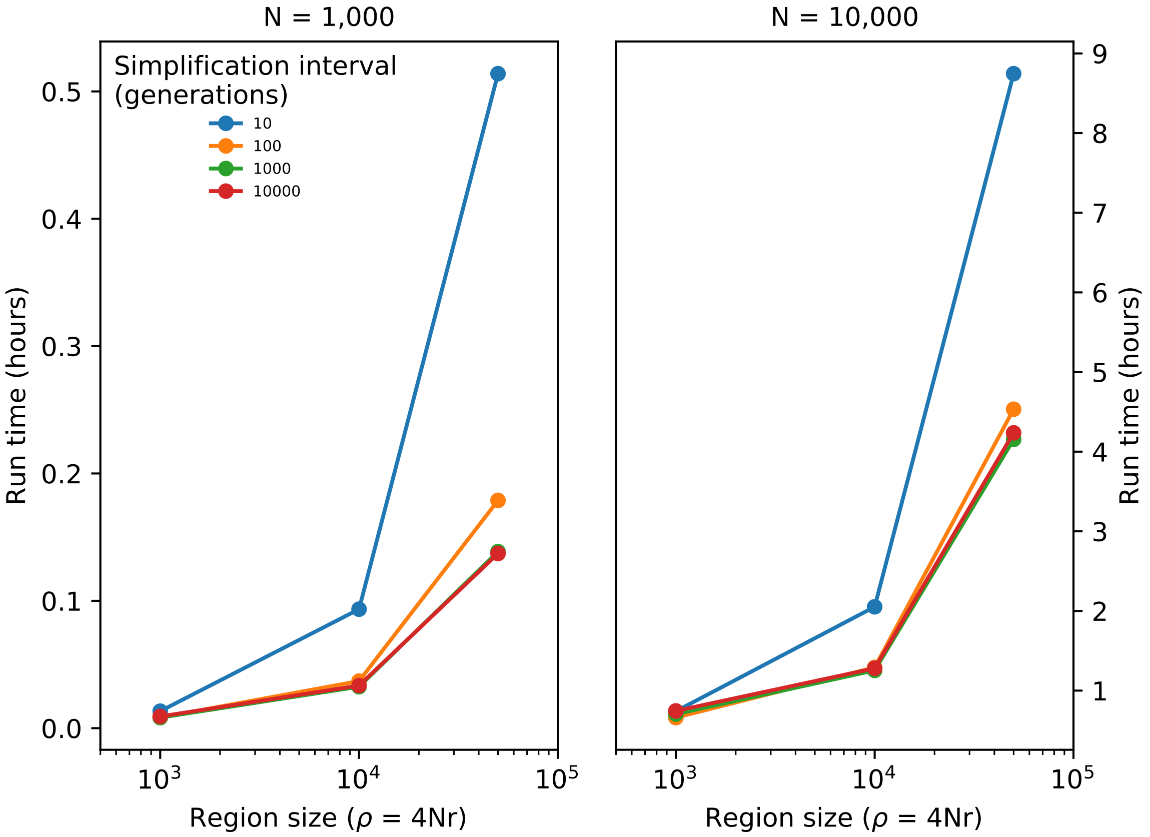

Figure C2: The effect of simplification interval on run times. A single replicate was done for the parameters shown in the figure. These simulations were done with the same selection parameters as in Figure 1.

D The `simuPOP` implementation

To use the methods with `simuPOP`, we created a python module (available at `https://github.com/ashander/ftprime`, version 0.0.6) that manages communication between the forward simulator and `tskit`. We implemented simulations in a Python script, usable as a command-line utility, taking input parameters for population size N and scaled recombination rate ρ. As for the `fwdpp` simulations above, we set scaled mutation rate $\theta = 4N\mu = \rho$ (where μ is the per-site mutation rate multiplied by the number of sites), and used a simplify interval of 1000 generations and simulated for $T = 10N$ generations in each simulation.

We coupled `simuPOP` to `tskit` using a Python class `RecombCollector` that 1) tracks time, and 2) collects and parses recombination information provided by the `Recombinator` class of `simuPOP`, adding them to a **NodeTable**() and **EdgeTable**() upon which simplification is performed every 1000 generations (using `tskit.sort_tables()` and `tskit.simplify_tables`). The tables are initialized using the

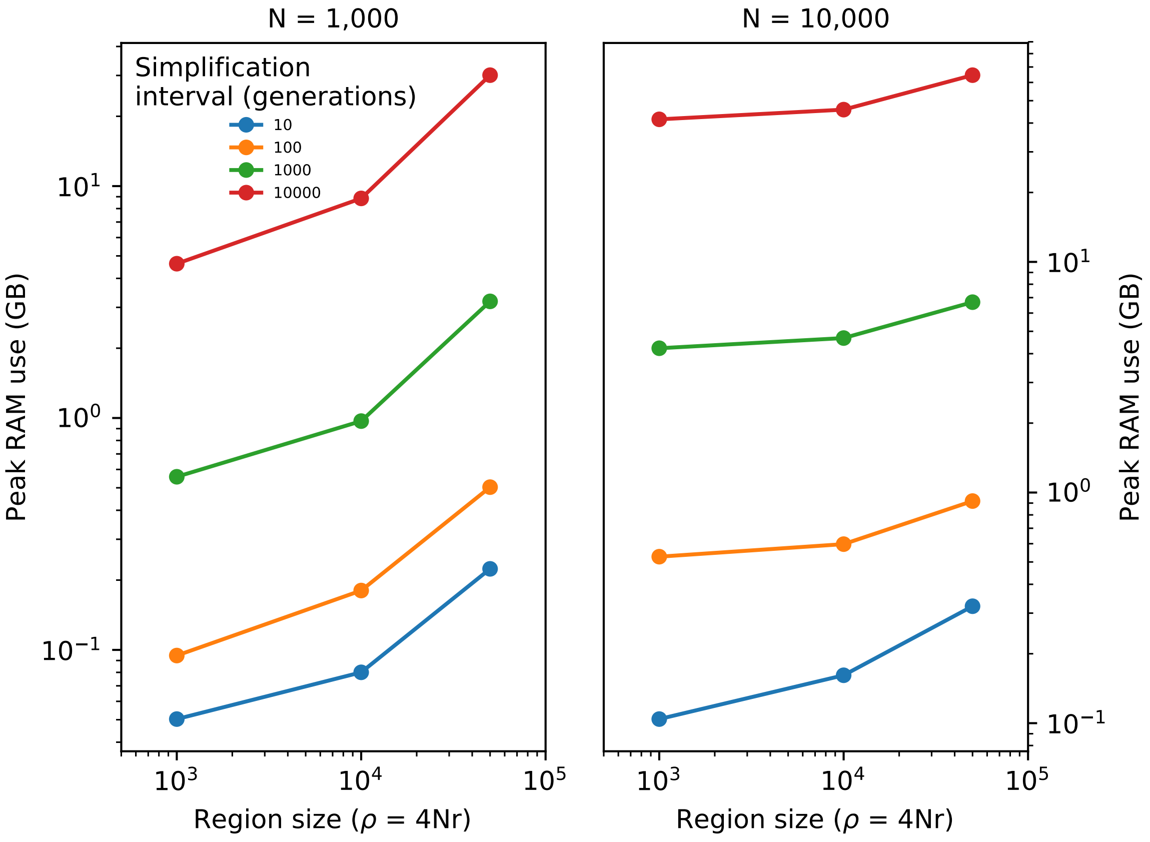

Figure C3: Peak RAM use for the simulations shown in Figure C2.

`TreeSequence` returned from `tskit.simulate(`$2N$`, Ne=`N`, recombination_rate=`$r/2$`, length=`n`)`. The `simuPOP.Population()` is initialized and tagged with individual IDs, a mapping of these IDs to the **NodeTable**() IDs is passed to an instance of `RecombCollector` and used internally to update the tables. The `simuPOP.Population()` is then evolved. Before mating: the internal time of the `RecombCollector` instance is updated. During mating: male and female parents are drawn randomly, recombination data is output by `simuPOP` to the `RecombCollector` instance. After mating: every 1000 generations the tables are simplified.

`RecombCollector` uses the Python class `ARGrecorder` internally to add data to the tables and perform simplification. This class contains the **NodeTable**() and **EdgeTable**() and also the mapping from (haploid) ids to ids in the tables. It also provides methods (`add_individual` and `add_record`) to append data to the tables. The only functions of `RecombCollector` not delegated to this internal class are computing haploid IDs from `simuPOP`'s diploid IDs and parsing recombination data into data suitable for use with use with `add_individual` and `add_record`; these functions are used to stream data into the tables as it is received by an instance of `RecombCollector`.

E Simplification algorithm

Here we provide a concrete implementation of the simplification algorithm. The algorithm uses a few simple structures besides the node and edge tables discussed earlier. As the algorithm needs to track the mapping

between input and output node IDs over specific genomic intervals, we use a **Segment**() type to track these individual mappings. Thus, a segment x has three attributes: x.left and x.right represent the genomic interval in the usual way, and x.node represents the output node ID that is assigned to this interval. These output node IDs correspond to colors in the “paint pot” analogy. The per-interval segments are assigned to input IDs via the $\mathcal{A}$ mapping, which is the main state required by the algorithm. Each element $\mathcal{A}[u]$ is a list of ancestral segments, describing the output node IDs mapped to input node ID u over a set of intervals. For each input node, we need to process the ancestral segments to find overlaps (and hence coalescences), and to update $\mathcal{A}$. We do this using a **PriorityQueue**() type, which maintains a list of ancestral segments sorted by left coordinate. For a priority queue Q, the operation Q.**push**(x) inserts the segment x into the queue, and Q.**pop**() removes and returns the segment in Q with the smallest left coordinate. To obtain the segment currently in the queue with the smallest left value we write $Q[0]$. Finally, note that to keep the exposition simple, the algorithm described here requires that the input node table is sorted by time since the parent's birth; but the version implemented in `tskit` makes different sortedness assumptions (described in the documentation).

Algorithm S. (*Simplify a tree sequence*). Input consists of a list S of sample IDs, a list $\mathcal{N}_I$ of nodes (ordered by birth time), a list $\mathcal{E}_I$ of edges, and the genome length, L. The output is a nodes $\mathcal{N}_O$, and list of edges $\mathcal{E}_O$.

S1. [Initialisation.] Set $\mathcal{A}_u \leftarrow$ **List**() for $0 \leq u < |\mathcal{N}_I|$. Then, for each u in S, set $v \leftarrow \mathcal{N}_O$.**addrow**($\mathcal{N}_I[u]$) and call $\mathcal{A}[u]$.**append**(**Segment**($0, L, v$)). Then set $Q \leftarrow$ **PriorityQueue**() and $u \leftarrow 0$.

S2. [Select parent edges.] Set $E \leftarrow \{e \in \mathcal{E}_I : e.\text{parent} = u\}$. Set $v \leftarrow -1$. If $|E| = 0$, go to S9.

S3. [Insert edge/ancestry intersections.] For each $e \in E$ and each $x \in \mathcal{A}[e.\text{child}]$, if $x.\text{right} > e.\text{left}$ and $e.\text{right} > x.\text{left}$, set $\ell \leftarrow \max(x.\text{left}, e.\text{left})$, $r \leftarrow \min(x.\text{right}, e.\text{right})$, $y \leftarrow$ **Segment**($\ell, r, x.\text{node}$) and call Q.**push**(y).

S4. [Find segments with minimum left coordinate.] Set $\ell \leftarrow Q[0].\text{left}$, $r \leftarrow L$ and $X \leftarrow \text{List}()$. Then, while $|Q| > 0$ and $Q[0].\text{left} = \ell$, set $x \leftarrow Q$.**pop**(), $r \leftarrow \min(r, x.\text{right})$ and call X.**append**(x). Afterwards, if $|Q| > 0$, set $r \leftarrow \min(r, Q[0].\text{left})$. If $|X| > 1$ go to S6.

S5. [No overlapping segments.] Set $x \leftarrow X[0]$ and $\alpha \leftarrow x$. If $|Q| > 0$ and $Q[0].\text{left} < x.\text{right}$, set $\alpha \leftarrow$ **Segment**($x.\text{left}, Q[0].\text{right}, x.\text{node}$), $x.\text{left} \leftarrow Q[0].\text{left}$ and call Q.**push**(x). Go to S8.

S6. [Overlap; new output node.] If $v = -1$, set $v \leftarrow \mathcal{N}_0$.**addrow**($\mathcal{N}_I[u]$).

S7. [Record edges.] Set $\alpha \leftarrow$ **Segment**(ℓ, r, v). Then for each $x \in X$ call $\mathcal{E}_O$.**addrow**($l, r, v, x.\text{node}$) and, if $x.\text{right} > r$, set $x.\text{left} \leftarrow r$ and call Q.**push**(x).

S8. [Left coordinate loop.] Call $\mathcal{A}[u]$.**append**(α). If $|Q| > 0$ return to S4.

S9. [Parent loop.] Set $u \leftarrow u + 1$. If $u < |\mathcal{N}_I|$, go to S2.

The algorithm begins in S1 by allocating an empty list to store ancestral segments for each of the $|\mathcal{N}_I|$ input nodes, and then creating the initial state for the samples in S. For each input sample with node ID u, we add a row in the output node table $\mathcal{N}_O$, and create a mapping for this new node v. After this initial state has been created, we then allocate our priority queue Q, and set the current input ID u to 0. Recall that the function $\mathcal{N}$.**addrow**(t) adds a new node to the node table $\mathcal{N}$ with birth time t, and returns the ID (i.e., index) of that new node in $\mathcal{N}$. So, the operation $v \leftarrow \mathcal{N}_O$.**addrow**($\mathcal{N}_I[u]$) looks up the birth time for input node ID u in $\mathcal{N}_I$, adds a new node with this time to $\mathcal{N}_O$, and stores the resulting (output) node ID in v.

To process ancestral information moving back up through the pedigree, steps S2–S9 consider each node u in turn (note we are assuming that nodes are ordered by time-since-birth). In S2 we first find all edges in the input where the parent is equal to our current input ID u, and then set the corresponding output ID v to -1. Then, in S3 we find all intersections between these input edges and the mapped ancestry segments of their child nodes. For each of these intersections we create a new segment y and insert it into the priority queue. (In the paint pot analogy, this stores in Q all segments of color that must be transferred to the ancestor with input ID u.)

After finding all overlaps and filling the priority queue with the resulting ancestry segments, we then need to process these segments. Steps S4–S8 loop over the segments in the queue, considering each in turn until the queue is empty. Step S4 builds a list X of all segments covering the current left-most coordinate (along with some bookkeeping to keep track of where our next right coordinate r is), removing them from Q. If there is only one element in X this means that we have no overlapping ancestral segments at this coordinate and we proceed to S5.

In this case, only a single segment covers the leftmost region of the segments, so there is no change in the mapping between input and output IDs, and the ancestry segment α is not changed (this is assigned to $\mathcal{A}_u$ in S8). (The color in this segment of the offspring can simply be transferred to the parent.) However, if other segments in Q intersect with x (since x is the current left-most segment, these have left coordinate less then x.right), then we can only transfer the portion of x up until it overlaps with other segments. Therefore, we change the left coordinate of x to this coordinate and reinsert it into Q where it will be processed again later, effectively cutting off (and propagating) the leftmost segment of x. After doing this, we proceed to S8, update the ancestry mapping for the input node u, and loop back to S4 if Q is not empty.

On the other hand, if there is more than one segment in X, we know that an overlap among the ancestral segments has occurred and we must update the output nodes and edges accordingly. In step S6 we first allocate a new output node v for input node u, if it has not been done already (we may have many overlapping segments along the genome). Step S7 then continues by creating a new ancestry segment α mapping the current interval to the output node v. We then iterate over all segments in X, adding the appropriate output edges and editing and reinserting the ancestry segment x into the priority queue if necessary, as before.

Python implementation of simplify (supplementary information)

```
"""
Python implementation of Algorithm S.

The example works by generating an initial TreeSequence
for a sample of 10 haplotypes using msprime. We then
simplify the node/edge table in that TreeSequence with
respect to the first three samples.

Requires msprime >= 0.6.0
"""
import heapq
import numpy as np

import msprime


class Segment(object):
    """
    An ancestral segment mapping a given node ID to a half-open genomic
    interval [left, right).
    """
    def __init__(self, left, right, node):
        assert left < right
        self.left = left
        self.right = right
        self.node = node

    def __lt__(self, other):
        # We implement the < operator so that we can use the heapq directly.
        # We are only interested in the left coordinate.
        return self.left < other.left

    def __repr__(self):
        return repr((self.left, self.right, self.node))


def simplify(S, Ni, Ei, L):
    """
    This is an implementation of the simplify algorithm described in Appendix A
    of the paper.
    """
    tables = msprime.TableCollection(L)
    No = tables.nodes
    Eo = tables.edges
    A = [[] for _ in range(len(Ni))]
    Q = []

    for u in S:
        v = No.add_row(time=Ni.time[u], flags=1)
```

```
        A[u] = [Segment(0, L, v)]

    for u in range(len(Ni)):
        for e in [e for e in Ei if e.parent == u]:
            for x in A[e.child]:
                if x.right > e.left and e.right > x.left:
                    y = Segment(max(x.left, e.left), min(x.right, e.right), x.node)
                    heapq.heappush(Q, y)
        v = -1
        while len(Q) > 0:
            l = Q[0].left
            r = L
            X = []
            while len(Q) > 0 and Q[0].left == l:
                x = heapq.heappop(Q)
                X.append(x)
                r = min(r, x.right)
            if len(Q) > 0:
                r = min(r, Q[0].left)

            if len(X) == 1:
                x = X[0]
                alpha = x
                if len(Q) > 0 and Q[0].left < x.right:
                    alpha = Segment(x.left, Q[0].left, x.node)
                    x.left = Q[0].left
                    heapq.heappush(Q, x)
            else:
                if v == -1:
                    v = No.add_row(time=Ni.time[u])
                alpha = Segment(l, r, v)
                for x in X:
                    Eo.add_row(l, r, v, x.node)
                    if x.right > r:
                        x.left = r
                        heapq.heappush(Q, x)

            A[u].append(alpha)

    # Sort the output edges and compact them as much as possible into
    # the output table. We skip this for the algorithm listing as it's pretty mundane.
    # Note: could be replaced with calls to squash_edges() and sort_tables()
    E = list(Eo)
    Eo.clear()
    E.sort(key=lambda e: (e.parent, e.child, e.right, e.left))
    start = 0
    for j in range(1, len(E)):
        condition = (
            E[j - 1].right != E[j].left or
            E[j - 1].parent != E[j].parent or
            E[j - 1].child != E[j].child)
```

```
        if condition:
            Eo.add_row(E[start].left, E[j - 1].right, E[j - 1].parent, E[j - 1].child)
            start = j
    j = len(E)
    Eo.add_row(E[start].left, E[j - 1].right, E[j - 1].parent, E[j - 1].child)

    return tables.tree_sequence()



def verify():
    """
    Checks that simplify() does the right thing, by comparing to the implementation
    in msprime.
    """
    for n in [10, 100, 1000]:
        ts = msprime.simulate(n, recombination_rate=1, random_seed=1)
        nodes = ts.tables.nodes
        edges = ts.tables.edges
        print("simulated for ", n)

        for N in range(2, 10):
            sample = list(range(N))
            ts1 = simplify(sample, nodes, edges, ts.sequence_length)
            ts2 = ts.simplify(sample)

            n1 = ts1.tables.nodes
            n2 = ts2.tables.nodes
            assert np.array_equal(n1.time, n2.time)
            assert np.array_equal(n1.flags, n2.flags)
            e1 = ts1.tables.edges
            e2 = ts2.tables.edges
            assert np.array_equal(e1.left, e2.left)
            assert np.array_equal(e1.right, e2.right)
            assert np.array_equal(e1.parent, e1.parent)
            assert np.array_equal(e1.child, e1.child)


if __name__ == "__main__":
    # verify()

    # Generate initial TreeSequence
    ts = msprime.simulate(10, recombination_rate=2, random_seed=1)
    nodes = ts.tables.nodes
    edges = ts.tables.edges

    # Simplify nodes and edges with respect to the following samples:
    sample = [0, 1, 2]
    ts1 = simplify(sample, nodes, edges, ts.sequence_length)
    print(ts1.tables)
```
